# PD93 Uncertainty Metric To Guide PICOS Assessment And Evidence Synthesis Planning For Joint Clinical Assessment Submission

**DOI:** 10.1017/S0266462324003404

**Published:** 2025-01-07

**Authors:** Hannah Marjolein van Schoubroeck, Sonja Kroep, Emanuele Arca, Elisabeth Fenwick

## Abstract

**Introduction:**

With the European Union Regulation on health technology assessment (HTA) approaching, there is concern about how to accommodate the large number of expected population, intervention, comparator, outcome(s), and study type (PICOS) questions and their uncertainty. Navigating uncertainty will prove essential to anticipate evidence requirements. This abstract presents preliminary results of the development of a metric that assesses uncertainty surrounding PICOS questions to eventually guide planning of evidence synthesis for joint clinical assessment (JCA) submission.

**Methods:**

The metric will consist of pillars representing overarching themes of uncertainty. Each pillar will contain several elements influencing uncertainty for proposed PICOS questions. This study was conducted in two phases. In phase one, targeted literature searches of peer-reviewed and gray literature were conducted to identify the overarching metric pillars. These pillars were then validated by expert opinion. Similar research methods were used in phase two to inform the content of each of the pillars. Here we present the findings for phase one.

**Results:**

The targeted literature review in phase one resulted in multiple candidate elements for the uncertainty pillars. Elements were selected based on expert opinion, resulting in five main pillars. These pillars were considered most valuable for the determination of uncertainty surrounding individual PICOS questions and were considered crucial to HTA acceptance. Phase one resulted in the following pillars: (i) indication and subpopulations; (ii) type of intervention and comparators; (iii) societal and patient unmet need; (iv) type and quality of evidence source informing evidence synthesis; and (v) methodology of evidence synthesis.

**Conclusions:**

This study lays the foundation for a metric to assess the uncertainty surrounding proposed PICOS questions for JCAs. The metric identifies existing uncertainties for PICOS questions, assesses potential issues for HTA acceptance, and guides possible evidence synthesis planning. A clear framework for anticipating uncertainty will prove essential in managing resources and expectations.

